# Cross-Modality Interaction Network for Equine Activity Recognition Using Imbalanced Multi-Modal Data †

**DOI:** 10.3390/s21175818

**Published:** 2021-08-29

**Authors:** Axiu Mao, Endai Huang, Haiming Gan, Rebecca S. V. Parkes, Weitao Xu, Kai Liu

**Affiliations:** 1 Department of Infectious Diseases and Public Health, Jockey Club College of Veterinary Medicine and Life Sciences, City University of Hong Kong, Hong Kong, China; axmao2-c@my.cityu.edu.hk (A.M.); haimigan@cityu.edu.hk (H.G.); 2 Department of Computer Science, City University of Hong Kong, Hong Kong, China; edhuang2-c@my.cityu.edu.hk (E.H.); weitaoxu@cityu.edu.hk (W.X.); 3 College of Electronic Engineering, South China Agricultural University, Guangzhou 510642, China; 4 Department of Veterinary Clinical Sciences, Jockey Club College of Veterinary Medicine and Life Sciences, City University of Hong Kong, Hong Kong, China; reparkes@cityu.edu.hk; 5 Centre for Companion Animal Health, Jockey Club College of Veterinary Medicine and Life Sciences, City University of Hong Kong, Hong Kong, China; 6 Animal Health Research Centre, Chengdu Research Institute, City University of Hong Kong, Chengdu 610000, China

**Keywords:** equine behavior, wearable sensor, deep learning, intermodality interaction, class-balanced focal loss

## Abstract

With the recent advances in deep learning, wearable sensors have increasingly been used in automated animal activity recognition. However, there are two major challenges in improving recognition performance—multi-modal feature fusion and imbalanced data modeling. In this study, to improve classification performance for equine activities while tackling these two challenges, we developed a cross-modality interaction network (CMI-Net) involving a dual convolution neural network architecture and a cross-modality interaction module (CMIM). The CMIM adaptively recalibrated the temporal- and axis-wise features in each modality by leveraging multi-modal information to achieve deep intermodality interaction. A class-balanced (CB) focal loss was adopted to supervise the training of CMI-Net to alleviate the class imbalance problem. Motion data was acquired from six neck-attached inertial measurement units from six horses. The CMI-Net was trained and verified with leave-one-out cross-validation. The results demonstrated that our CMI-Net outperformed the existing algorithms with high precision (79.74%), recall (79.57%), F1-score (79.02%), and accuracy (93.37%). The adoption of CB focal loss improved the performance of CMI-Net, with increases of 2.76%, 4.16%, and 3.92% in precision, recall, and F1-score, respectively. In conclusion, CMI-Net and CB focal loss effectively enhanced the equine activity classification performance using imbalanced multi-modal sensor data.

## 1. Introduction

The behavior of horses provides rich insight into their mental and physical status and is one of the most important indicators of their health, welfare, and subjective state [1]. However, behavioral monitoring for animals, to date, largely relies on manual observations, which are labor-intensive, time-consuming, and prone to subjective judgments of individuals [1]. The use of sensors and machine learning is well-established in monitoring gait change [2], and for lameness detection as part of the equine veterinary examination, increasing the accuracy of identification of subtle lameness, which is one of the most expensive health issues in the equine industry [3,4]. Therefore it is of significant importance to investigate and develop an automatic, objective, accurate, and quantifiable measurement system for equine behaviors. Such a system will allow caretakers to identify variations in the animal behavioral repertoire in real-time, decreasing the workloads in veterinary clinics and improving the husbandry and management of animals [5,6].

Over recent decades, automated animal activity recognition has been studied widely with the aid of various sensors (e.g., accelerometers, gyroscopes, and magnetometers) and the use of machine learning techniques. For instance, a naïve Bayes (NB) classifier was applied to recognize horse activities (e.g., eating, standing, and trotting) using triaxial acceleration and obtained 90% classification accuracy [7]. Four classifiers including a linear discriminant analysis (LDA), a quadratic discriminant analysis (QDA), a support vector machine (SVM), and a decision tree (DT) were utilized to detect dog behaviors (e.g., galloping, lying on chest, and sniffing) based on accelerometer and gyroscope data, and the results revealed that the sensor placed on the back and collar yielded 91% and 75% accuracy at best, respectively [8]. A random forest (RF) algorithm was applied to categorize cow activities using triaxial acceleration and gained high classification accuracy with 91.4%, 99.8%, 88%, and 99.8% for feeding, lying, standing, and walking events, respectively [9]. In horses, the use of receiver-operating characteristic curve analysis classified standing, grazing, and ambulatory activities with a sensitivity of 94.7–97.7% and a specificity of 94.7–96.8% [10]. However, to classify animal behaviors accurately using these machine learning methods, feature extraction and method selection are often conducted manually and separately, which requires expert domain knowledge and easily induces feature engineering issues [11]. Moreover, handcrafted features often fail to capture general and complex features, resulting in low generalization ability, i.e., these extracted features perform well in recognizing the activities of some subjects but badly for others.

Along with the recent advances in internet technology and fast graphics processing units, various deep learning approaches have been increasingly and successfully adopted in animal activity recognition with wearable sensors. Classification models based on deep learning achieve automatic feature learning through data driving and subsequent animal activity recognition. For example, feed-forward neural networks (FNNs) and long short-term memory (LSTM) models were applied to automatically recognize cattle behaviors (e.g., feeding, lying, and ruminating) using data collected from inertial measurement units (IMUs) [12,13]. Convolutional neural networks (CNNs), which accurately capture local temporal dependency and scale invariance in signals, were developed in automated equine activity classification based on triaxial accelerometer and gyroscope data [1,14,15]. FilterNet, presented based on CNN and LSTM architectures, was adopted to classify important health-related canine behaviors (e.g., drinking, eating, and scratching) using a collar-mounted accelerometer [16].

However, multi-modal data fusion has not been well handled when different sensors are used simultaneously in existing studies. Multi-modal data with different characteristics are often simply processed using common fusion strategies such as early fusion, feature fusion, and result fusion [17]. The early fusion strategy used in previous studies [12,13], i.e., extracting the same features without distinction of modalities, often caused interference between multi-modal information due to their distribution gap [18]. The result fusion scheme was suboptimal since rich modality information was gradually compressed and lost in separate processes, ignoring the intermodality correlations. As a better choice, the feature fusion strategy fuses the intermediate information of multiple modalities, which avoids the distribution gap problem and achieves intermodality interaction simultaneously [19,20]. However, feature fusion is often limited to linear fusion (e.g., simple concatenation and addition) and fails to explore deep multi-modality interactions and achieve complementary-redundant information combinations between multiple modalities [17].

In addition, the collected sensor datasets often present class imbalance problems due to the inconsistent frequency and duration of each activity resulting from specific animal physiology. Deep learning methods trained on imbalanced datasets tend to be biased toward majority classes and away from minority classes, which easily causes poor modal generalization ability and high classification error rates for rare categories [21]. Commonly used methods on imbalanced datasets mainly involve two techniques, namely, resampling and reweighting. Resampling attempts to sample the data to obtain an evenly distributed dataset, e.g., oversampling and undersampling [22]. However, oversampling and undersampling come with high potential risks of overfitting and information loss, respectively [21]. Reweighting is more flexible and convenient by directly assigning a weight for the loss function per training sample to alleviate the sensitivity of the model to data distribution [23]. This method is further divided into class-level and sample-level reweighting. The former, such as cost-sensitive (CS) loss [24] and class-balanced (CB) loss [25], depends on the prior category frequency, while the latter, such as focal loss [26] and adaptive class suppression (ACS) loss [27], relies on the network output confidences of each instance. In addition, CB focal loss, combining a CB term with a modulating factor, effectively focuses on difficult samples and considers the proportional impact of effective numbers per class simultaneously [25].

To improve the recognition performance for equine activities while tackling the abovementioned challenges, we have developed a cross-modality interaction network (CMI-Net) which achieved a good classification performance in our previous work [28], and a CB focal loss [25] was adopted to supervise the training of CMI-Net. The CMI-Net consisted of a dual CNN trunk architecture and a joint cross-modality interaction module (CMIM). Specifically, the dual CNN trunk architecture extracted modality-specific features for accelerometer and gyroscope data, respectively, and the CMIM based on attention mechanism adaptively recalibrated the importance of the elements in the two modality-specific feature maps by leveraging multi-modal knowledge. The attention mechanism has been widely utilized in different tasks using multi-modal datasets such as RGB-D images [17,29]. It has also been adopted to focus on important elements along with channels and spatial dimensions of the same input feature [30,31]. The favorable performance presented in these studies with the attention mechanism indicated the rationality of our proposed CMIM. In our method, softmax cross-entropy (CE) loss was initially used to supervise the training of CMI-Net. However, softmax CE loss suffered from inferior classification performance, especially for monitory classes [23]. In contrast, CB focal loss, by adding a CB term to focal loss, focuses more on minor-class samples and hard-classified samples and can alleviate the class imbalance problem. Therefore, a CB focal loss [25] was also adopted. In this study, the CMI-Net was trained based on an extensively labeled dataset [32] to automatically recognize equine activities including eating, standing, trotting, galloping, walking-rider (walking while carrying a rider), and walking-natural (walking with no rider). The leave-one-out cross-validation (LOOCV) method was applied to test the generalization ability of our model, and the results were then compared to the existing algorithms. The main contributions of this paper can be summarized as follows:

- We proposed a CMI-Net involving a dual CNN trunk architecture and a joint CMIM to improve equine activity recognition performance using accelerometer and gyroscope data. The dual CNN trunk architecture comprised a residual-like convolution block (Res-LCB) which effectively promoted the representation ability and robustness of the model [33]. The CMIM based on attention mechanism enabled CMI-Net to capture complementary information and suppressed unrelated information (e.g., noise, redundant signals, and potentially confusing signals) from multi-modal data.
- We devised a novel attention module, i.e., CMIM, to achieve deep intermodality interaction. The CMIM combined spatial information from two-stream feature maps using basic CNN to produce two spatial attention maps with respect to their importance, which could adaptively recalibrate temporal- and axis-wise features in each modality. To the best of our knowledge, the attention mechanism was employed for the first time in animal activity recognition based on multi-modal data yielded by multiple wearable sensors.
- We adopted a CB focal loss to supervise the training of CMI-Net to mitigate the influence of imbalanced datasets on overall classification performance. The CB focal loss can pay more attention not only to samples of minority classes, diminishing their influence from being overwhelmed during optimization, but also to samples that are hard to distinguish. As far as we know, this is the first time the CB focal loss has been utilized in animal activity recognition based on imbalanced datasets.
- Experiments performed verified the effectiveness of our proposed CMI-Net and CB focal loss. In particular, the experimental results demonstrated that our CMI-Net outperformed the existing algorithms in equine activity recognition with the precision of 79.74%, recall of 79.57%, F1-score of 79.02%, and accuracy of 93.37%, respectively.

## 2. Materials and Methods

### 2.1. Data Description

**Figure 1:**
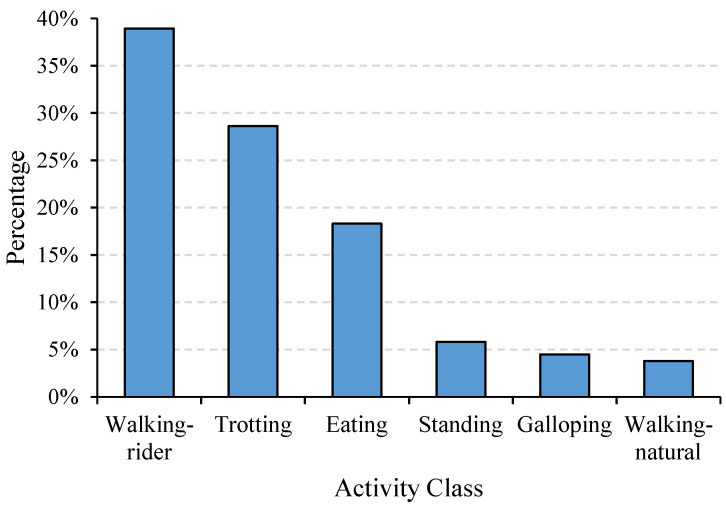
Histogram of class distribution.

The dataset used in this study was a public dataset created by Kamminga et al. [32]. In this dataset, more than 1.2 million 2 s data samples were collected from 18 individual equines using neck-attached IMUs. The sampling rate was set to 100 Hz for both the triaxial accelerometer and gyroscope and 12 Hz for the triaxial magnetometer. The majority of the samples were unlabeled, but data from six equines and six activities including eating, standing, trotting, galloping, walking-rider, and walking-natural were labeled extensively (87,621 2 s samples in total) and were used to classify equine activities in previous studies [7,34]. In this study, data from the triaxial accelerometer and gyroscope among the 87,621 samples were exploited separately, forming up to two tensors with a size of 1 × 3 × 200 for each sample. As demonstrated in Figure 1, the activities of eating, standing, trotting, galloping, walking-rider, and walking-natural occupied 18.32%, 5.84%, 28.62%, 4.50%, 38.94%, and 3.80% of the total sample number, respectively, producing a maximum imbalance ratio of 10.25. In addition, the input sample of each axis per sensor modality was normalized by removing the mean and scaling to unit variance, which can be formulated as follows:

(1) $${\tilde{S}}_{i}=\frac{S_{i}-\mu_{i}}{\sigma_{i}},$$

where $S_{i}$ denotes all samples of a particular axis per sensor modality (i.e., X-, Y-, and Z-axis of the accelerometer, and X-, Y-, and Z-axis of the gyroscope), ${\tilde{S}}_{i}$ denotes all normalized samples, and $\mu_{i}$ and $\sigma_{i}$ denote mean and standard deviation values in each axis per sensor modality, respectively.

### 2.2. Cross-Modality Interaction Network

**Figure 2:**
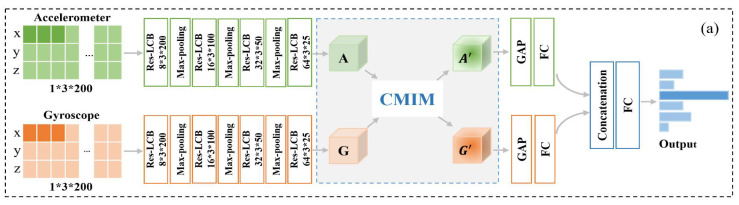

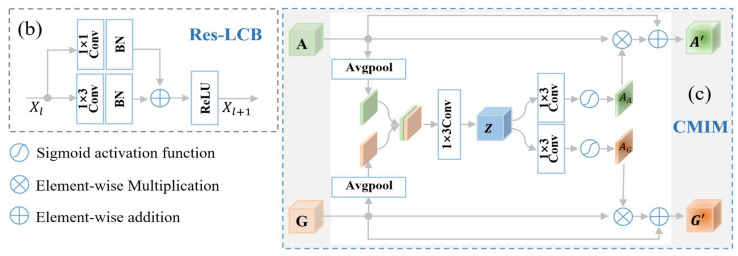
The architecture of our proposed cross-modality interaction network (CMI-Net). (**a**) Our proposed CMI-Net. The size of the feature maps is marked after every residual-like convolution block (Res-LCB) layer. Here, “A” and “G” denote the modality-specific features for the accelerometer and gyroscope, respectively, and “$A^{\prime }$” and “$G^{\prime }$” denote the refined features after modality interaction. “GAP” and “FC” are the global average-pooling layer and fully connected layer, respectively. (**b**) Res-LCB and (**c**) cross-modality interaction module (CMIM).

Our proposed CMI-Net, where accelerometer and gyroscope data were fed into two CNN branches (represented by CNN_acc_ and CNN_gyr_) separately, is shown in Figure 2a. The dual CNN was constructed to extract modality-specific features and concatenate these features before the final dense layer. To achieve deep interaction between the two-modality data and capture the complementary information and suppress unrelated information from them, a joint CMIM was designed and inserted in the upper layer. The details are described below.

#### 2.2.1. Dual CNN Trunk Architecture

The CNN_acc_ and CNN_gyr_ contained four convolution blocks, three max-pooling layers, one global average-pooling layer, and one fully connected layer, followed by concatenation and one joint fully connected layer. Inspired by the residual unit in the deep residual network that behaves like ensembles and has smaller magnitudes of responses [33], to promote the representation ability and robustness of the model, we designed a Res-LCB, as demonstrated in Figure 2b. The definition is given below.

(2) $$X_{l+1}=RELU\left(Conv^{1\times 1}\left(X_{l}\right)⊕Conv^{1\times 3}\left(X_{l}\right)\right),$$

where $X_{l}$ and $X_{l+1}$ denote feature maps in the l and l+1 layers, respectively, $Conv^{1\times 1}\left(•\right)$ and $Conv^{1\times 3}\left(•\right)$ represent 1 × 1 and 1 × 3 convolution operations, respectively, ⊕ denotes the elementwise addition, and *RELU* (•) denotes the rectified linear unit activation function [35].

#### 2.2.2. Cross-Modality Interaction Module

Inspired by the multi-modal transfer module that recalibrates channel-wise features of each modality based on multi-modal information [36] and the convolutional block attention module that focuses on the spatial information of the feature maps [30], we devised a CMIM based on an attention mechanism to adaptively recalibrate temporal- and axis-wise features in each modality by utilizing multi-modal information. The detailed CMIM is illustrated in Figure 2c.

Let $A\in R^{C\times H\times W}$ and $G\in R^{C\times H\times W}$ represent the features at a given layer of CNN_acc_ and CNN_gyr_, respectively. Here, *C*, *H*, and *W* denote the channel number and spatial dimensions of features. Specifically, *H* and *W* correspond to the axial and temporal signals, respectively. The CMIM receives *A* and *G* as input features. We first applied average-pooling operations along channels of the input features, generating two spatial maps. These two maps were then concatenated and mapped into a joint representation $Z\in R^{C^{\prime }\times H\times W}$. The operation was shown as follows:

(3) $$Z=RELU\left(Conv^{1\times 3}\left(\left[Avgpool\left(A\right),\;Avgpool\left(G\right)\right]\right)\right),$$

where $C^{\prime }$ denotes the channel number of feature *Z*, *Avgpool* (•) denotes the average-pooling operation, and [•] denotes the concatenation operation. Furthermore, two spatial attention maps $A_{A}\in R^{1\times H\times W}$ and $A_{G}\in R^{1\times H\times W}$ were generated through two independent convolution layers with a sigmoid function σ(•) using the joint representation *Z*:

(4) $$A_{A}=\sigma \left(Conv^{1\times 3}\left(Z\right)\right),\;A_{G}=\sigma \left(Conv^{1\times 3}\left(Z\right)\right),$$

$A_{A}$ and $A_{G}$ were then used to recalibrate the input features, generating two final refined features, i.e., $A^{\prime }\in R^{C\times H\times W}$ and $G^{\prime }\in R^{C\times H\times W}$:

(5) $$A^{\prime }=A⊗A_{A}⊕A,\;G^{\prime }=G⊗A_{G}⊕G,$$

where ⊗ denotes the elementwise multiplication. Specifically, each convolution operation under this study was followed by a batch normalization operation. The increases in channel numbers and decreases in spatial dimensions were implemented through Res-LCB and max-pooling operations, respectively.

### 2.3. Optimization

As the most widely utilized loss in the multiclass classification task, softmax CE loss was applied to optimize the parameters of CMI-Net. The formulation of softmax CE loss was defined as

(6) $$L_{CE}\left(z\right)=-\sum_{i=1}^{C}y_{i}log\left(p_{i}\right)$$

(7) $$\mathrm{with}\;p_{i}=\frac{e^{z_{i}}}{\sum_{j=1}^{C}e^{z_{j}}},$$

where *C* and $z=\left[z_{1},\ldots ,z_{C}\right]$ are the total number of classes and the predicted logits of the network, respectively. In addition, $y_{i}\;\ldots \;\left\{0,1\right\}$, 1 ≤i ≤C is the one-hot ground-truth label. However, the models based on softmax CE loss often suffer from inferior classification performance, especially for monitory classes, due to the imbalanced data distribution [23]. Therefore, we further introduced an effective loss function to supervise the training of CMI-Net and alleviate the class imbalance problem, namely, CB focal loss.

CB focal loss, which added the CB term to the focal loss function, focused more on not only samples of minority classes, diminishing their influence from being overwhelmed during optimization, but also samples that were hard to distinguish. The CB term was related to the inverse effective number of samples per class, and focal loss added a modulating factor to the sigmoid CE loss to reduce the relative loss for well-classified samples and focused more on difficult samples. The CB focal loss was presented as

(8) $$L_{CB_{FL}}\left(z\right)=\frac{1}{E_{n_{y}}}L_{FL}\left(z\right)=-\frac{1-\beta }{1-\beta^{n_{y}}}\sum_{i=1}^{C}{\left(1-p_{i}^{t}\right)}^{\gamma }log\left(p_{i}^{t}\right)$$

(9) $$\mathrm{with}\;p_{i}^{t}=\frac{1}{1+e^{-z_{i}^{t}}},$$

(10) $$z_{i}^{t}=\{\begin{array}{ll} z_{i}, & if\;i=y.\\ -z_{i}, & \mathrm{otherwise}. \end{array},$$

where $n_{y}$ and $E_{n_{y}}$ represent the actual number and the effective number of the ground-truth label y, respectively. The hyperparameter *β* ∈ [0, 1) controlled how fast $E_{n_{y}}$ grows as $n_{y}$ increases, and *γ* ≥ 0 smoothly adjusted the rate at which easy samples were down-weighted [26]. The value of *β* was set to 0.9999, and the search space of the hyperparameter *γ* was set to {0.5, 1.0, 2.0} [25] in this study. In particular, CB loss and focal loss rebalanced the loss function based on class-level and sample-level reweighting, respectively. Thus, we also utilized class-level reweighted losses, including cost-sensitive cross-entropy loss (CS_CE loss) [24], class-balanced cross-entropy loss (CB_CE loss) [25], and sample-level reweighted losses, including focal loss [26] and adaptive class suppression loss (ACS loss) [27], to validate the effectiveness of the CB focal loss.

### 2.4. Evaluation Metrics

The comprehensive performance of the equine activity classification model was indicated by the following four evaluation metrics, which are defined in Equations (11)–(14). Each indicator value was multiplied by 100 as the result to reflect the difference in indicator values more clearly.

(11) $$\mathrm{Precision}=\frac{\mathrm{TP}}{\mathrm{TP}+\mathrm{FP}},$$

(12) $$\mathrm{Recall}=\frac{\mathrm{TP}}{\mathrm{TP}+\mathrm{FN}},$$

(13) $$F1-\mathrm{Score}=\frac{2\mathrm{TP}}{2\mathrm{TP}+\mathrm{FP}+\mathrm{FN}},$$

(14) $$\mathrm{Accuracy}=\frac{\mathrm{TP}+\mathrm{TN}}{\mathrm{TP}+\mathrm{TN}+\mathrm{FP}+\mathrm{FN}},$$

where TP, FP, TN, and FN are the number of true positives, false positives, true negatives, and false negatives, respectively. In particular, the overall precision, recall, and F1-score were calculated by using a macro-average [37].

### 2.5. Implementation Details

To attain subject-dependent results, the LOOCV method was used, in which four subjects were chosen for training, one for validation, and one for testing each time and rotated in a circular manner. During training, the loss function was added by an L2 regularization term with a weight decay of 0.1 to avoid overfitting. An Adam optimizer with an initial learning rate of 1 × 10^−4^ was employed, and the learning rate decreased by 0.1 times every 20 epochs. The number of epochs and batch size were set to 100 and 256, respectively. The best model with the highest validation accuracy was saved and verified using test data. To evaluate the classification performance of our CMI-Net, we compared it against various existing methods, including three machine learning methods (i.e., NB, DT, and SVM) and two deep learning methods used in equine activity recognition (i.e., CNN and ConvNet7) [14,15], based on the same public dataset. Specifically, the hand-crafted features used in machine learning were the same as those used by Kamminga et al. [7]. To further explore the performance of our CMIM, we ran the network without CMIM and with it inserted after the 1st, 2nd, and 3rd max-pooling layers to obtain four different variants, i.e., Variant0, Variant1, Variant2, and Variant3, respectively. The softmax CE loss was used as the loss function for all variants. All experiments were executed using the PyTorch framework on an NVIDIA Tesla V100 GPU. The developed source code will be available at https://github.com/Max-1234-hub/CMI-Net from 1 September 2021.

## 3. Results and Discussion

Overall, experiments conducted on the public dataset demonstrated that our proposed CMI-Net outperformed the existing algorithms. Ablation studies were then carried out to verify the effectiveness of CMIM and that applying the CMIM in the upper layer of CMI-Net could obtain better performance. Different loss functions were adopted to validate that CB focal loss performed better than any class-level or sample-level reweighted loss used alone, and it effectively improved the overall precision, recall, and F1-score, although the overall accuracy decreased due to the imbalanced dataset used. Furthermore, recognition performance analysis was presented to help us probe the predicted performance on each activity using our CMI-Net with CB focal loss. The details are described as follows.

### 3.1. Comparison with Existing Methods

The comparison results of our CMI-Net with three machine learning methods (i.e., NB, DT, and SVM) and two deep learning methods (i.e., CNN and ConvNet7) [14,15] are illustrated in Table 1. The results revealed that the CMI-Net with softmax CE loss outperformed the machine learning algorithms with higher precision, recall, F1-score, and accuracy of 79.74%, 79.57%, 79.02%, and 93.37%, respectively. The reason for this superior performance was the convolution and pooling operations in CNN, which could achieve automated feature learning and aggregate more complex and general patterns without any domain knowledge [38]. The other CNN-based method [15] obtained inferior precision of 72.07% and accuracy of 82.94% compared to DT and SVM. This result is consistent with the “No Free Lunch” theorem [39] because this CNN-based method [15] was developed using leg-mounted sensor data. In addition, our CMI-Net with softmax CE loss performed better than ConvNet7 [14], which obtained lower precision, recall, F1-score, and accuracy of 79.03%, 77.79%, 77.90%, and 91.27%, respectively. This was attributed to the ability of our architecture to effectively capture the complementary information and inhibit unrelated information of multi-modal data through deep multi-modality interaction. In addition, CMI-Net with CB focal loss (*γ* = 0.5) enabled the values of precision, recall, and F1-score to increase by 2.76%, 4.16%, and 3.92%, respectively, compared with CMI-Net with softmax CE loss. This revealed that the adoption of CB focal loss effectively improved the overall classification performance.

### 3.2. Ablation Study

#### 3.2.1. Evaluation of CMIM

To explore the effectiveness of CMIM and the impact of its position in the network on classification performance, the results corresponding to four different variants are shown in Table 2. Our proposed CMI-Net with softmax CE loss showed superior performance to Variant0 (i.e., the network without CMIM), indicating the effective performance of our interaction module. Variant1, Variant2, and Variant3 (i.e., networks with CMIM inserted after 1st, 2nd, and 3rd max-pooling layer, respectively) did not perform better in terms of precision and recall compared with Variant0, which obtained precision and recall values of 79.02% and 77.09%, respectively. This might be explained by the fact that modality-specific features learned in the shallow layer were simple and contained noise, which interfered with the process by which CMIM learned complex intermodality correlations, leading to poor predictions [40]. In addition, our architecture obtained the best performance since it applied the CMIM after a deeper layer, which enabled the network to discover more discriminative patterns and suppress irrelevant variations more effectively [41].

**Figure 3:**
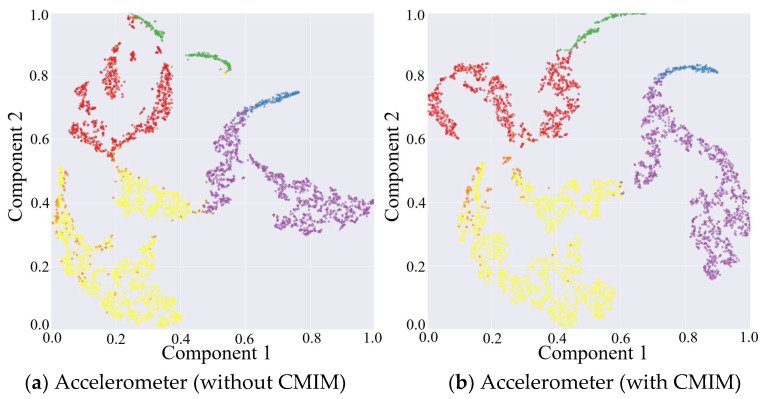

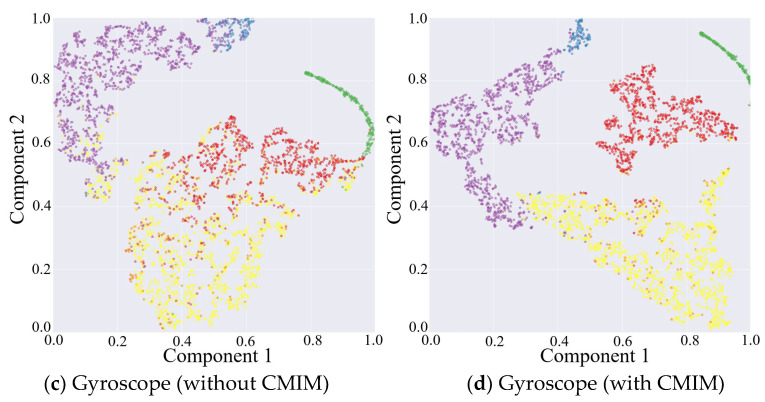
Embedding visualization of the features extracted from triaxial accelerometer and gyroscope data under network without and with CMIM, respectively.

**Figure 4:**
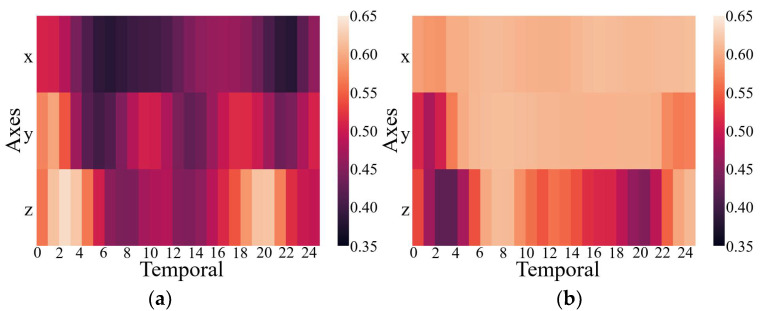
Attention maps for features extracted from the triaxial accelerometer (**a**) and gyroscope (**b**) data.

The results above have proven that the inclusion of the CMIM in the network provided quantifiable improvements in identification performance. This was also reflected in the qualitative visualization of the embeddings and the corresponding clusters in Figure 3, with the help of t-distributed stochastic neighbor-embedding (t-SNE), a technique for visualizing high-dimensional data by giving each data point a location in a two- or three-dimensional map [42]. Figure 3 shows the two-dimensional embedded features from the part test dataset after the fully connected layers of both CNN branches under the network without and with CMIM by using the t-SNE technique with an init of ‘pca’ and perplexity of 30. Comparing the left and right columns in Figure 3, it can be observed that more compact clusters were generated under the network with CMIM by reducing the intraclass distance and enlarging the interclass distance. The core technical point was that the joint interaction module enabled adaptive amplification of salient features and suppression of unrelated features based on information from two-modality data. To further provide insights into its contribution, we presented two spatial attention maps for features extracted from the triaxial accelerometer and triaxial gyroscope data (Figure 4). As illustrated in Figure 4, the value per pixel represented the contribution degree corresponding to each temporal period and each axis, and it was adaptively recalibrated through intermodality interaction. Therefore, both quantitative and qualitative findings reinforced the suitability of our proposed CMI-Net to tasks using two-modality sensor data.

#### 3.2.2. Evaluation of CB Focal Loss

To study the effect of CB focal loss on the optimization of CMI-Net, we show the quantitative performance in Table 3 and explore the sensitivity of its hyperparameter *γ*. CMI-Net with CB focal loss (*γ* = 0.5) achieved the best precision of 82.50%, recall of 83.73%, and F1-score of 82.94%. This indicated that CB focal loss was beneficial to the improvement of classification performance when the modulation strength was controlled appropriately, whereas negative effects occurred if the value of *γ* was too large or too small.

**Figure 5:**
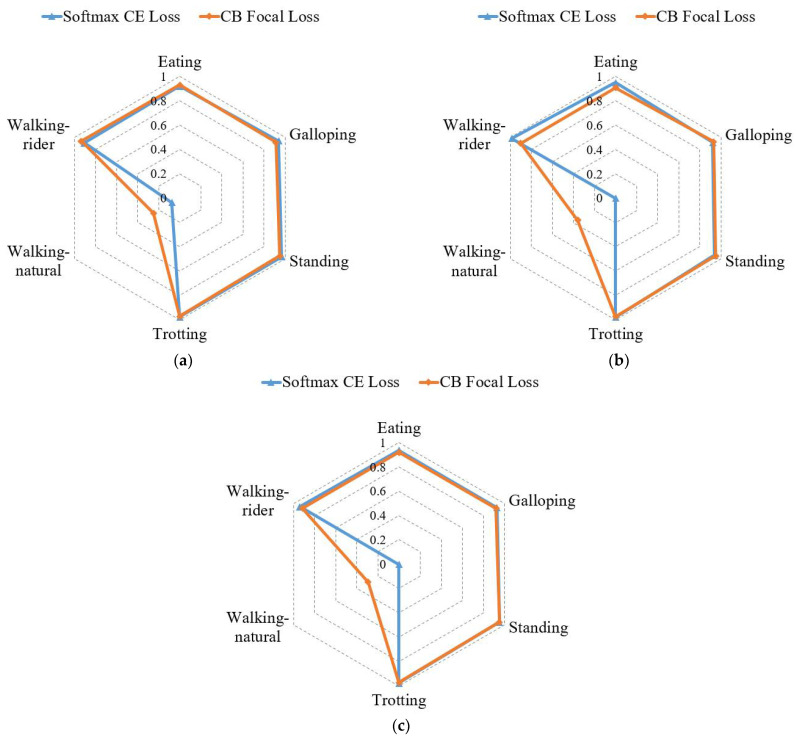
Precision (**a**), recall (**b**), and F1-score (**c**) comparison of each activity under softmax cross-entropy (CE) loss and class-balanced (CB) focal loss.

To provide further insight into the influence of CB focal loss (*γ* = 0.5) on the classification performance, we present the classification results of each activity under CMI-Net with CB focal loss and softmax CE loss, respectively, in Figure 5. It shows that precision, recall, and F1-score of the walking-natural were significantly improved, while other activities varied slightly when using CB focal loss. This explained that the overall classification performance increased mainly due to the increase in walking-natural, as it focused more on difficult samples and samples of minority classes. However, the overall accuracy of CMI-Net with CB focal loss decreased by 2.69% (Table 3), which was related to the different variations of recall values in different activities and the current imbalanced dataset. In particular, the overall accuracy could also be presented as the weighted average of the recall value for each activity according to the sampling frequency of each activity. As shown in Figure 5, the recall increases were 35.92% for walking-natural, 1.17% for standing, and 0.91% for galloping, and the recall decreases were 8.41% for walking-rider, 4.26% for eating, and 0.36% for trotting when using CB focal loss. It can be observed that all activities with increased recall belonged to the minority class, while the remaining activities with decreased recall belonged to the majority class, resulting in a decrease in overall accuracy. Thus, it is necessary to collect a more balanced dataset in the future.

**Figure 6:**
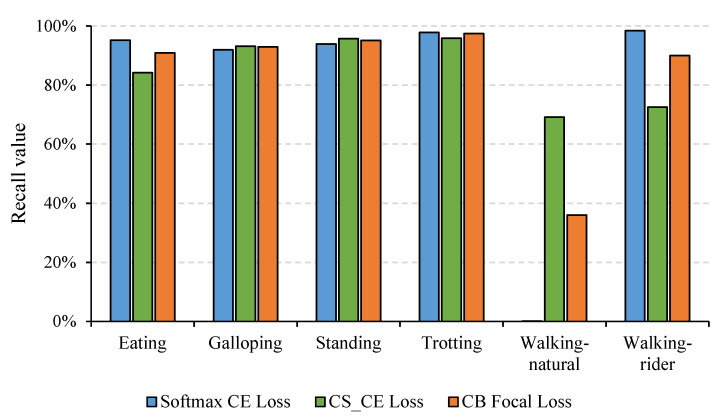
Recall of different activities under different loss functions including softmax CE loss, cost-sensitive cross-entropy (CS_CE) loss, and CB focal loss.

In addition, experiments under different loss functions were conducted to verify the effectiveness of the CB focal loss, as illustrated in Table 4. The contrasting losses mainly included CS_CE loss, CB_CE loss, focal loss, and ACS loss, as mentioned in the “Optimization” section. We found that CB focal loss combining CB loss and focal loss performed better than any of them used alone, which indicated that adding the CB term to the focal loss function improved the overall classification performance on the imbalanced dataset. In addition, the precision, recall, and F1-score of CS_CE loss and CB focal loss increased by different degrees, while both accuracies decreased compared with softmax CE loss. Specifically, the accuracy was only 83.79%, although the recall reached the highest value of 85.11%. This was because the recall of walking-rider was only 72.49%, although that of walking-natural was 69.16% (Figure 6). This result further verified that decreased accuracy occurred when using balancing techniques on the imbalanced dataset. In addition, we found that the recall of majority classes decreased while that of minority classes increased when using CS_CE loss and CB focal loss (Figure 6). This result revealed that both losses effectively focused on the samples of minority classes during training, but it is inevitable that more samples in majority classes were misclassified as minority classes so that overall accuracy would decrease.

### 3.3. Classification Performance Analysis

**Figure 7:**
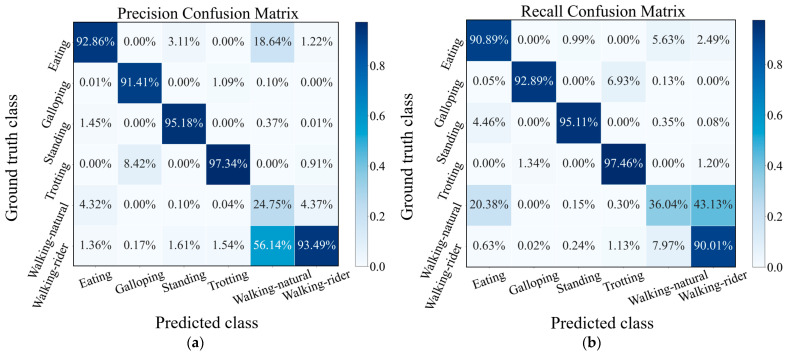
Precision (**a**) and recall (**b**) confusion matrix of CMI-Net with CB focal loss (*γ* = 0.5).

**Figure 8:**
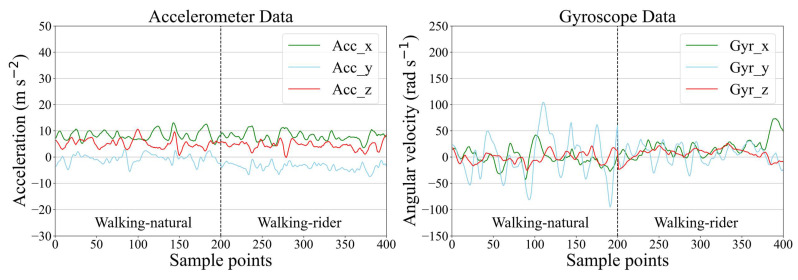
Example of accelerometer and gyroscope data for walking-natural and walking-rider.

In Figure 7, we show the precision and recall confusion matrix aggregating the classification results under 6-fold cross-validation when using CMI-Net with CB focal loss (*γ* = 0.5). Both precision and recall values of all activities had more than 90% accuracy (i.e., the precision and recall for eating were 92.86% and 90.89%, for galloping were 91.41% and 92.89%, for standing were 95.18% and 95.11%, for trotting were 97.34% and 97.46%, and for walking-rider were 93.49% and 90.01%, respectively), except for the walking-natural activity, which only obtained low precision and recall (Figure 7). This low classification precision and recall occurred for two main reasons. The first reason was class imbalance. Walking-natural as the minority class in the dataset only occupied 3.8%, which was much less than the 38.94% occupation of majority class walking-rider, which easily caused the model to be biased toward the majority classes and resulted in poor minority class recognition performance. The second reason was severe confusion with other activities, especially eating and walking-rider activities. As shown in Figure 7, 18.64% and 56.14% of the samples predicted to be class walking-natural had ground truth classes eating and walking-rider, respectively. In addition, 20.38% and 43.13% of the samples with ground truth class walking-natural were misclassified as class eating and walking-rider, respectively. This was because, during eating, the horse was slowly walking so that some samples of eating might contain walking activity [32]. The movement patterns of walking-natural and walking-rider were very similar, which interfered with the learning ability of the network for these two behavioral characteristics (Figure 8). It also revealed that there was no major variability in equine walking patterns in the presence or absence of a rider. This was consistent with a previous study that found no major changes in equine limb kinematics, although the extension of the thoracolumbar region increased during walking with a rider compared with non-ridden walking [43]. In addition, there was confusion between galloping and trotting activities with misclassification of 6.93% of galloping as trotting. This might be related to the misinterpretation by the annotator during labeling, as it was not always clear when the activity transitions occurred [32]. Additionally, a sample rate of 100Hz may limit the distinction in the transition between trotting and cantering or galloping.

### 3.4. Limitations and Future Works

The first limitation of our proposed method is that our model was trained on a public dataset that contained only six labeled activities, i.e., eating, standing, trotting, galloping, walking-rider, and walking-natural. Indeed, there are some other activities such as head shaking, scratch biting, rubbing, and rolling, all of which, although infrequent, are physiologically critical to equine health and welfare, and should have been labeled and included in the dataset. Due to the missing of these infrequent activities in the dataset, inevitably, as a typical open-set recognition problem [44], these unlabeled activities that occur in real behavior monitoring scenarios will be easily misclassified as the six defined activities, resulting in loss of some key information. Thus, as a next step to further improve classification performance for equine activities, we will investigate some feasible techniques such as classification-reconstruction learning and weightless neural networks [44,45,46] to enable our activity classifiers to not only accurately classify the defined classes appearing in training but also effectively deal with unlabeled ones generated in practice.

The second limitation is that the algorithms we developed and adopted in this study were based on supervised learning, which relied on a large number of annotated samples. Data annotation is a labor-intensive and time-consuming task, and well-annotated data is often limited as reflected by the fact that we can only find one public dataset for equine activities. With regard to the found dataset [32], in fact, there are still vast amounts of unlabeled samples that can be used to alleviate the overfitting problem and improve the generalization ability of models. Thus, how we can best use the unlabeled samples becomes a key. To this point, our work can be further expanded toward the direction of semi-supervised learning to sufficiently exploit these unlabeled data. For instance, we may first train models on the existing and well-labeled data and then apply the trained models to conduct predictions for unlabeled data. The one-hot predictions can serve as pseudo labels for those high-confidence samples, which, along with the original labels, can then be further used to train the model iteratively until the unlabeled data no longer changes.

## 4. Conclusions

In this study, we developed a CMI-Net involving a dual CNN trunk architecture and a joint CMIM to improve equine activity classification performance. The CMI-Net effectively captured complementary information and suppressed unrelated information from multiple modalities. Specifically, the dual CNN architecture extracted modality-specific features, and the CMIM recalibrated temporal- and axis-wise features in each modality by utilizing multi-modal knowledge and achieved deep intermodality interaction. To alleviate the class imbalance problem, a CB focal loss was leveraged for the first time to supervise the training of CMI-Net, which focused more on the difficult samples and samples of minority classes during optimization. The results revealed that our CMI-Net with softmax CE loss outperformed the existing methods, and the adoption of CB focal loss effectively improved the precision, recall, and F1-score while slightly decreasing the accuracy. In addition, ablation studies demonstrated that applying the CMIM in the upper layer of CMI-Net could obtain better performance since high-level features contained more general patterns. CB focal loss also performed better than any class-level or sample-level reweighted losses used alone. In short, the favorable classification performance indicated the effectiveness of our proposed CMI-Net and CB focal loss.

## Figures and Tables

**Table 1:** Classification performance comparison with existing methods. The best two results for each metric are highlighted in bold.

Methods	Precision (%)	Recall (%)	F1-Score (%)	Accuracy (%)
Machine learning				
Naïve Bayes	70.90	72.41	69.42	76.60
Decision tree	75.67	73.90	74.35	88.83
Support vector machine	73.92	71.30	72.19	89.65
Deep learning				
CNN [15]	72.07	76.91	73.42	82.94
ConvNet7 [14]	79.03	77.79	77.90	**91.27**
Our methods ^#^				
CMI-Net + softmax CE loss	**79.74**	**79.57**	**79.02**	**93.37**
CMI-Net + CB focal loss (*γ* = 0.5) *	**82.50**	**83.73**	**82.94**	90.68

^#^ CMI-Net: cross-modality interaction network; CE: cross-entropy; CB: class-balanced; * the *γ* of value is 0.5, which could refer to Table 3.

**Table 2:** Performance comparison of our CMI-Net with its variants. The best results for each metric are highlighted in bold.

Methods ^&^	Precision (%)	Recall (%)	F1-Score (%)	Accuracy (%)
Variant0 ^#^	79.02	77.09	76.88	91.76
Variant1 *	78.18	77.07	77.40	92.17
Variant2 *	77.50	78.44	77.91	92.92
Variant3 *	78.36	76.94	77.02	92.62
CMI-Net + softmax CE loss	**79.74**	**79.57**	**79.02**	**93.37**

^&^ denotes all networks presented in this table were trained using softmax CE loss; ^#^ denotes the network without a cross-modality interaction module (CMIM); * denotes the network where the CMIM was inserted after the 1st, 2nd, and 3rd max-pooling layers, respectively.

**Table 3:** Performance comparison between softmax CE loss and CB focal loss with different *γ*. The best results for each metric are highlighted in bold.

Loss Functions	Precision (%)	Recall (%)	F1-Score (%)	Accuracy (%)
Softmax CE Loss (baseline)	79.74	79.57	79.02	**93.37**
CB focal loss (*γ* = 0.1)	81.31	83.60	81.97	89.57
CB focal loss (*γ* = 0.5)	**82.50**	**83.73**	**82.94**	90.68
CB focal loss (*γ* = 1)	80.42	82.03	81.05	89.89
CB focal loss (*γ* = 2)	78.92	78.48	77.97	91.05

**Table 4:** Classification performance comparison with different loss functions. The best two results for each metric are highlighted in bold.

Loss Functions ^#^	Precision (%)	Recall (%)	F1-Score (%)	Accuracy (%)
Softmax CE loss	79.74	79.57	79.02	**93.37**
Class-level				
CS_CE loss [24]	**80.47**	**85.11**	**79.91**	83.79
CB_CE loss [25]	75.35	75.70	75.47	90.61
Sample-level				
Focal loss [26]	78.84	77.99	78.25	**93.30**
ACS loss [27]	77.03	76.54	76.60	92.05
CB focal loss (*γ* = 0.5)	**82.50**	**83.73**	**82.94**	90.68

^#^ CS_CE: cost-sensitive cross-entropy; CB_CE: class-balanced cross-entropy; ACS: adaptive class suppression.

## Abbreviations

ACS	Adaptive class suppression
CB	Class-balanced
CB_CE	Class-balanced cross-entropy
CE	Cross-entropy
CMIM	Cross-modality interaction module
CMI-Net	Cross-modality interaction network
CNN	Convolutional neural network
CS_CE	Cost-sensitive cross-entropy
DT	Decision tree
FN	False negative
FNNs	Feed-forward neural networks
FP	False positive
IMUs	Inertial measurement units
LDA	Linear discriminant analysis
LOOCV	Leave-one-out cross-validation
LSTM	Long short-term memory
NB	Naïve Bayes
QDA	Quadratic discriminant analysis
Res-LCB	Residual-like convolution block
RF	Random forest
SVM	Support vector machine
TN	True negative
TP	True positive
t-SNE	t-distributed stochastic neighbor embedding
